# Whipple’s Disease: A Challenging Diagnosis

**DOI:** 10.7759/cureus.51991

**Published:** 2024-01-10

**Authors:** Beatriz Porteiro, Ana Rita Freitas, Filipa Nunes, Marinela Major, Frederico Batista

**Affiliations:** 1 Internal Medicine, Hospital Prof. Doutor Fernando Fonseca, Amadora, PRT; 2 Oncology, Hospital Prof. Doutor Fernando Fonseca, Amadora, PRT

**Keywords:** deep vein thrombosis (dvt), reactive lymphadenitis, polyarthralgia, tropheryma whipplei, whipple's disease

## Abstract

Whipple’s disease (WD) is a chronic multisystemic infection caused by *Tropheryma whipplei*. It is a rare condition with a wide spectrum of clinical presentations, necessitating a high clinical suspicion to arrive at the diagnosis. We present the case of a 65-year-old woman who experienced chronic, intermittent, and migratory polyarthralgia, weight loss, anorexia, and pyrosis. She was admitted due to bilateral deep vein thrombosis (DVT). She exhibited lymphadenopathy without hepatosplenomegaly, and lymph node biopsy revealed reactive lymphadenitis with intrahistiocytic bacilli that reacted positively to periodic acid-Schiff staining. This led to the suspicion of WD, which was subsequently confirmed through small bowel biopsies. She initiated treatment with ceftriaxone and experienced rapid clinical improvement. WD poses a diagnostic challenge. The signs and symptoms are often nonspecific and can result in misdiagnosis as a rheumatic or neoplastic disease. The presentation with DVT, while unusual, has been reported as a manifestation of WD.

## Introduction

*Tropheryma whipplei* is a Gram-positive bacillus widely distributed in the environment. Although George Whipple first documented Whipple’s disease (WD) in 1907, *T. whipplei* was not identified until 1991 through a polymerase chain reaction (PCR) assay. Not all individuals carrying the agent develop infection [1]. In fact, WD is rare, with an incidence reported to be less than one case per 1,000,000 people [2].

It typically affects middle-aged Caucasian males. Primary infections can manifest as symptomatic episodes, resembling acute infectious illnesses such as gastroenteritis, pneumonia, and bacteremia, and, in most cases, lead to bacterial clearance and seroconversion. However, in some instances, the bacteria persist within the host, leading to chronic disease or asymptomatic carriage depending on genetic predisposition [3-4].

Classic WD is characterized by gastrointestinal symptoms, including steatorrhea, abdominal pain, and weight loss, as well as joint-related symptoms like arthralgias [5]. WD can affect nearly any organ system, with constitutional symptoms, cardiac involvement, neurological issues, and ocular symptoms being among the most common. It displays a wide array of manifestations, making it a "great mimic" of various diseases [6]. Thrombotic manifestations are seldom described in the literature and are associated with increased monocyte procoagulant activity [7-9]. In this report, we present a case of venous thrombosis as the initial manifestation of WD.

## Case presentation

A 65-year-old woman was admitted to our emergency department with a one-week history of pain and edema in both legs. Approximately three months prior to admission, she reported significant weight loss (5 kg, equivalent to 8% of her total body weight), anorexia, constipation, and worsening joint pain. Her medical history included a ten-year follow-up in rheumatology for intermittent inflammatory axial and peripheral joint pain, unresponsive to painkillers or mesotherapy; she had been diagnosed with a presumptive diagnosis of osteoarthritis. In recent months, her primary care physician had ordered blood tests that showed anemia, attributed to anemia of chronic disease (Table 1). She denied experiencing abdominal pain, nausea, diarrhea, vomiting, fever, headache, or any neurological symptoms.

Upon admission, physical examination revealed painless axillary lymph nodes, each measuring approximately 1 cm with elastic consistency, and bilateral non-pitting edema of the lower limbs. She did not exhibit hepatosplenomegaly or joint swellings, and the neurological examination was unremarkable.

**Figure 1:**
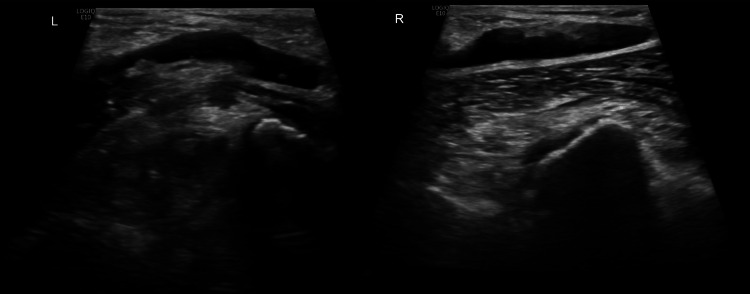
Echo-Doppler ultrasound of the lower limbs Heterogeneous luminal filling and lack of compressibility on the left (L) and right (R) popliteal veins, aspects compatible with bilateral deep vein thrombosis

Laboratory tests showed normocytic normochromic anemia with hyperferritinemia and cytocholestasis with hypoalbuminemia, along with elevated inflammatory markers (Table 1). An Echo-Doppler ultrasound of the lower limbs confirmed the diagnosis of bilateral popliteal venous thrombosis (Figure 1). Investigations for prothrombotic conditions (antithrombin III, factor V Leiden, lupus anticoagulant, and anti-cardiolipin IgG and IgM) were conducted and yielded negative results.

**Figure 2:**
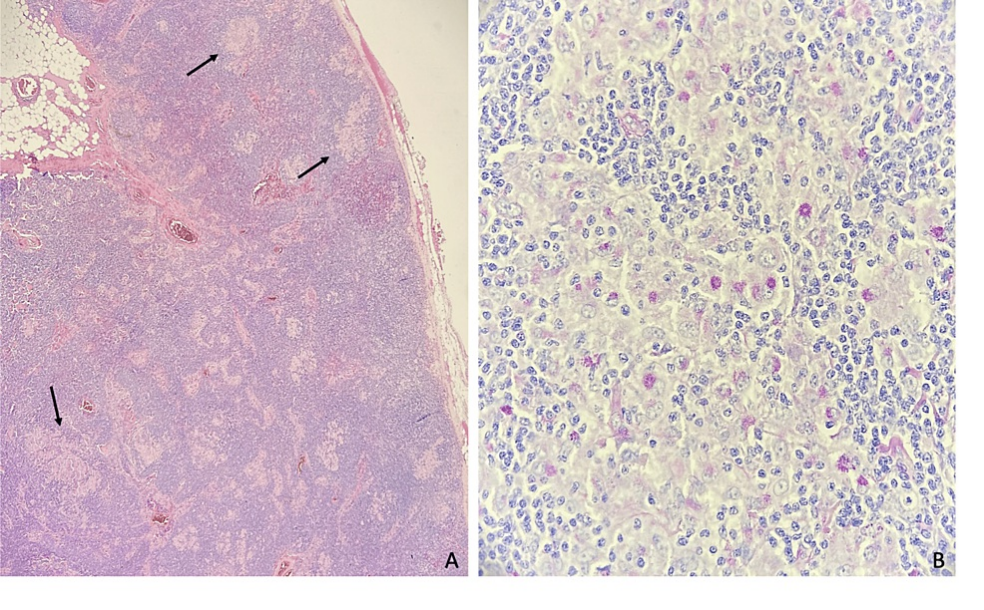
Anatomopathological study of lymph node (A) Lymph node exhibiting distorted architecture characterized by a significant number of histiocytes, at times aggregated and forming non-necrotizing granulomas (black arrows). (B) Histochemical examination revealed threadlike structures, resembling bacilli, located intrahistiocytically, and these structures tested positive for PAS (Periodic Acid-Schiff) and PAS-diastase. No morphological or immunohistochemical evidence of neoplastic tissue was identified. Images kindly provided by Dr. Joaquim Tinoco (Anatomical Pathology Service of Hospital Prof. Doutor Fernando Fonseca)

Based on these findings, treatment with enoxaparin at a dose of 1 mg/kg twice a day (BID) was initiated, and further investigations regarding a paraneoplastic syndrome were undertaken. An abdominal ultrasound revealed hepatic steatosis. The CT scan showed multiple lymph nodes in various groups: axillary, internal mammary, cardiophrenic, retroperitoneal, and mesenteric. Additionally, there was adenopathy in the hepatic hilum and fat densification near the hepatic hilum and the cephalic trunk, suggestive of a lymphoproliferative disease. An excisional axillary lymph node biopsy was performed, revealing granulomatous, non-necrotizing reactive lymphadenitis, likely of infectious origin. The presence of intrahistiocytic bacilli that reacted to periodic acid-Schiff staining raised the possibility of WD (Figure 2).

**Figure 3:**
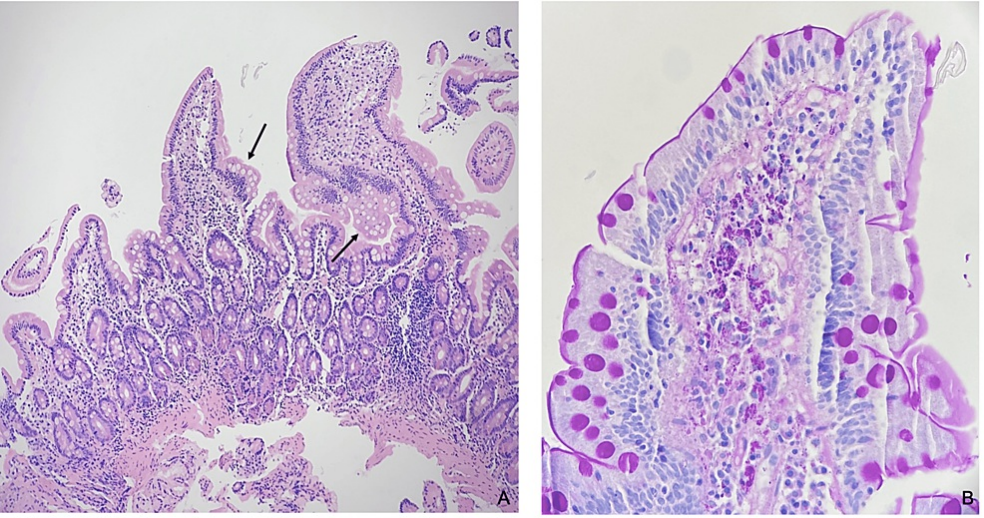
Histopathological study of small intestine biopsies (A) Proximal intestine with relatively preserved crypt architecture, alongside mucosal expansion in certain villi due to the presence of xanthochromic macrophages (black arrows), H&E 4x. (B) In the histochemical analysis, intracytoplasmic bacilli that tested positive for PAS (Periodic Acid-Schiff) and PAS-D (Diastase) were identified. The Ziehl-Neelsen staining showed negative results, PAS-D 10x Images kindly provided by Dr. Joaquim Tinoco (Anatomical Pathology Service of Hospital Prof. Doutor Fernando Fonseca)

To confirm the diagnosis, an upper digestive endoscopy was performed. The macroscopic examination did not reveal any abnormalities, but the histological findings from duodenal biopsies and the isolation of *T. whipplei* by polymerase chain reaction (PCR) confirmed the presence of WD (as shown in Figure 3). Antibiotic treatment with ceftriaxone at a dose of 2g once a day was initiated for 15 days, followed by cotrimoxazole 800+160 mg twice a day for one year.

One year after the diagnosis, the patient is asymptomatic, with no joint pain, and she has regained her usual weight. Her blood tests are within normal limits (Table 1), and she underwent repeated intestinal biopsies that showed no abnormalities.

**Table 1:** Blood tests before and after Whipple’s disease treatment MCV - mean corpuscular volume, MCH - mean corpuscular hemoglobin, ESR - erythrocyte sedimentation rate, CRP - C-reactive protein, aPTT - activated partial thromboplastin time, TP - prothrombin time, AST -  aspartate transaminase, ALT - alanine transaminase, ALP - alkaline phosphatase, GGT - gammaglutamyltranspeptidase, LDH - lactate dehydrogenase

Laboratory Results	Before	After	Normal Range
Hemoglobin	10.7 g/L	12 g/L	12–15 g/L
MCV	85.3 fL	85.3 fL	81–97 fL
MCH	26.3 pg	26.3 pg	27–32 pg
Leukocytes	7.1 × 10^9^/L	5 × 10^9^/L	4.5–11.5 10^9^/L
Platelets	385 × 10^9^/L	260 × 10^9^/L	150–450 g/L
ESR	57 mm/H	12 mm/H	0–20 mm/H
CRP	1.12 mg/dL	0.17 mg/dL	<0.5 mg/dL
aPTT	25″	25″	20.6–29.5″
TP	11.6″	11.6″	9.7–11.8″
AST	125 IU	25 IU	13–31 IU
ALT	161 IU	15 IU	13–32 IU
ALP	280 IU	80 IU	25–100 IU
GGT	143 IU	14 IU	4–32 IU
LDH	204 IU	162 IU	200–480 IU
Total bilirubin	0.25 mg/dL	0.16 mg/dL	0.2 mg/dL
Ferritin	300 ng/mL	150 ng/mL	10–291 ng/mL
Iron	79 µg/dL		33–193 µg/dL
Transferrin	131 mg/dL		200–360 mg/dL
Transferrin saturation	42%		20–50%
Albumin	3.25 g/dL	4.11 g/dL	3.5–5 g/dL
Antibodies anti-HIV 1 and 2	Negative		

## Discussion

WD is a chronic multisystemic disease that remains challenging to recognize. The multiplicity of presentations contributes to the extended period between the onset of symptoms and diagnosis [8], in our case over 10 years.

Genetic alterations, including patients with HLA associations (HLA alleles DRB1*13 and DQB1*06), IL-16 gene polymorphisms, and polymorphisms that enhance T-helper-2 (Th2) cell activity, as well as immunosuppression, are associated with WD [9]. In the duodenum, there is an accumulation of the bacillus within infected macrophages. These infected macrophages undergo alternative activation (M2) with inadequate maturation of antigen-presenting cells and impaired phagosome maturation, which promotes bacillus survival due to an excess of interleukin (IL)-10 and IL-16 and an absence of IL-12 and interferon (IFN)-γ. This process is responsible for inhibiting the stimulation of antigen-specific Th1 cells and stimulating the proliferation of regulatory T cells (Treg), leading to further reductions in IFN-γ and IL-12 levels. Collectively, these factors contribute to the spread and persistence of the infection [4, 10-11]. WD is responsible for chronic B-cell activation, which can lead to hypergammaglobulinemia and potentially contribute to the development of lymphoma: in our case, the activation of B cells is documented by enlarged lymph nodes. This phenomenon resembles infections such as Helicobacter spp. infection [12].

The activation of the coagulation system in WD is not well described. WD is characterized by chronic systemic inflammation, which presumably disrupts the hemostatic balance, leading to a prothrombotic state. The production of inflammatory cytokines induces lymphocyte infiltration through the endothelium, followed by focal inflammation and the production of IL-6, IL-8, and TNF, which can contribute to vasculitic processes. *T. whipplei* has the ability to infect and activate monocytes, further contributing to thrombosis, especially in areas where there is an imbalance in Virchow's triad [3,11,13].

The rarity of WD, its broad spectrum of symptoms, and the absence of specific clinical features collectively contribute to the challenge of diagnosis, often resulting in delayed recognition.

In patients presenting with symptoms suggestive of WD, the diagnosis is typically confirmed through the examination of duodenal biopsy samples obtained during upper endoscopy. A hallmark of the disease is the expansion of the lamina propria with numerous foamy macrophages containing PAS-positive globular cytoplasmic inclusions [14]. A definitive diagnosis is provided by a PCR assay targeting *T. whipplei*'s 16S ribosomal genes, applicable whether the PAS test is positive or negative. Additionally, it aids in the identification of extra-intestinal involvement [5, 10].

The treatment regimen for WD typically involves extended antibiotic therapy, with a consensus favoring the use of antibiotics that can penetrate the blood-brain barrier [8], given that the central nervous system (CNS) is the most common site for disease recurrence. Clinical improvement is usually observed within the first 7 to 21 days after initiating antibiotic therapy [15], as was observed in our patient. However, symptoms such as arthropathy, neurological issues, and cardiac symptoms may take several weeks to improve [16].

The treatment protocol includes induction therapy of ceftriaxone at a dose of 2 g intravenously (IV) once a day or meropenem at a dose of 1 g IV three times a day for two weeks, followed by long-term therapy with co-trimoxazole at a dose of 960 mg orally twice a day, or doxycycline at a dose of 200 mg orally per day in combination with hydroxychloroquine at a dose of 600 mg orally per day for one year [16]. Antibiotic resistance has been reported, but the resistance mechanisms are not yet known [10].

It is recommended to obtain repeated duodenal biopsy samples with PCR testing after treatment to determine when to discontinue antibiotics and to identify any potential relapses.

Relapses in WD are uncommon but tend to occur shortly after discontinuation of antibiotic therapy. There is a suggestion of a genetic predisposition for infection, as some patients experience more than one relapse and may benefit from lifelong antibiotic treatment [10].

## Conclusions

With this case, we underscore the potential for misdiagnosis of WD, which remains challenging to identify clinically, especially when it presents atypically with extraintestinal manifestations. This leads to concerns about delayed diagnosis in daily practice. Additionally, we contribute to the literature by presenting another case that initially manifested as deep vein thrombosis. While similar cases have been reported, further research is needed to better understand the pathophysiology involved in thrombus formation in such cases.
